# The Potential Influence of Organic Food Consumption and Intention-Behavior Gap on Consumers’ Subjective Wellbeing

**DOI:** 10.3390/foods9050650

**Published:** 2020-05-18

**Authors:** Diana Ismael, Angelika Ploeger

**Affiliations:** Specialized Partnerships in Sustainable Food Systems and Food Sovereignty, University of Kassel, 37213 Kassel, Germany; a.ploeger@uni-kassel.de

**Keywords:** intention-behavior gap, organic food consumption, subjective wellbeing, conventional food, food test, physical, emotional, social and intellectual dimensions, German consumers

## Abstract

This paper applied a self-administered survey to investigate the impact of organic food behavior and the intention-behavior gap in organic food consumption (OIBG) on consumers’ subjective wellbeing including physical, emotional, social and intellectual dimensions. The survey was carried out with 385 consumers. Furthermore, the study conducted a food test to explore the different impacts of organic and conventional food samples on the mental and physical conditions of consumers’ wellbeing applying a psychological questionnaire. The food test took place in a sensory lab with a panel of 63 untrained German consumers. The research findings demonstrated a positive impact of the organic food consumption on consumers’ subjective wellbeing, while no negative impact of OIBG has been perceived. Moreover, during the food test, consumers distinguished no differences between the impact of organic and conventional stimuli on their mental and physical status. Understanding how consumers perceive the impact of organic food consumption on their wellbeing is one important aspect. However, in the interest of narrowing the OIBG, it is more important to understand how consumers perceive the impact of this gap on their daily-life wellbeing.

## 1. Introduction

Wellbeing is considered a broad concept that involves a more holistic view of life. It is identified to be a multidimensional concept that is based on several interconnected dimensions [1,2]. This multidimensional nature of wellbeing has been addressed earlier by Hettler [3] and Roscoe [4], who have classified six interdependent dimensions for wellbeing: physical, emotional, social, intellectual, occupational and spiritual. As food is well-known to have great influence on our health, mood and emotions as well as our satisfaction with life, it is seen to have a strong impact on subjective wellbeing [5,6,7,8,9]. It is noticeable in the literature that a wide range of definitions of food-related wellbeing is emerging. For instance, Block et al. [10] defined food-related well-being as “a positive psychological, physical, emotional, and social relationship with food at both individual and societal levels.” Recent food studies explained that food-related wellbeing is mainly based on four common dimensions represented by the physical, intellectual, emotional and social dimensions, in addition to overall satisfaction with life [2,4,7,8,11,12].

Consumers’ perception of food-related wellbeing can have a bigger effect on their food habits and may help to address a more holistic assessment of a food product than overall liking, health or environmental concerns. Accordingly, subjective wellbeing has become a major focus in food marketing, sensory and consumer research. Researchers have become more concerned about the effect of food intake on subjective wellbeing [6,10]. According to Diener and Rayan [13], a better understanding of how to improve subjective wellbeing in the food context can provide opportunities to promote a higher quality of food lifestyle and help to develop successful food policies.

Organic food is highly related to the concept of subjective wellbeing as it is proven to be associated with health, emotions and social wellness [5,14,15,16,17,18,19,20,21,22,23,24,25]. Health is seen as the leading factor behind green choices [26] and one of the factors that give organic food an advantage over conventional food. However, current food manufacturers seek not only to market a “healthy product” but rather to provide consumers with food products that improve their wellbeing and make them feel better and satisfied [27,28].

As wellbeing is considered one of the most important aspects that people aim to achieve in their lives, understanding the association between subjective wellbeing and organic food can contribute to a better comprehension of the consumer’s behavior and final choice of organic food [29]. Investigating how organic food impacts consumers’ health and emotions has been an interesting core for plenty of previous and current research. However, studying the effect of organic food on subjective wellbeing has been scarce comparing to health and emotional aspects [30,31]. Thus, a more clear understanding of this relationship is currently needed [29,32].

Moreover, research demonstrates that consumers usually express great enthusiastic attitudes towards organic food, though their actual buying behavior falls short to these attitudes [33,34,35,36,37,38,39]. This disparity between their intentions to buy organic products and the relatively lower level of actual purchasing is acknowledged as the intention-behavior gap in organic food consumption (OIBG). The organic food market is considered a strong representative of this gap. This gap has been previously attributed to different reasons such as high prices, limited availability, lack of trust or lack of knowledge [22,34,37,40]. The effect of the intention-behavior gap on consumer’s subjective wellbeing, including the physical, emotional, social and intellectual dimensions, as far as we are aware, has not been addressed in literature yet.

During the progress of developing wellbeing scales, studies have focused on the effects of specific food products [12] or food in general [41]. Nonetheless, few have looked at the effect of organic food in particular, and none, to the best of our knowledge, has considered consumers’ perception of the effect of OIBG on their subjective wellbeing. Thus, this work imposes the questions of what is the potential influence of organic food consumption and, more importantly, the intention-behavior gap on consumers’ subjective wellbeing. How would OIBG make consumers feel in general and what are the barriers behind this gap from the consumers’ perception? 

Furthermore, in addition to applying self-administered questionnaires to measure how food influence the different dimensions of wellbeing [7,30,41,42], food tests were also conducted to explore consumers’ mental and physical changes after food intake [43,44]. Geier et al. [43,44] developed a psychological questionnaire to observe the effect of food consumption on consumers’ mental and psychological state related to their wellbeing status. Their work proved that untrained consumers can report mental and physical changes caused by distinct food types. Our study examines, based on Geier et al. test, whether consumers would feel any differences in their mental or physical states between organic and conventional food samples during a food test. This work is the first to focus on how organic and conventional food could differ in their effect on subjective wellbeing during a food consumption experience.

As wellbeing is a broad concept that combines health, emotions, social and other aspects of life, this work aims to understand how consumers perceive the effect of organic food consumption and mainly OIBG on some of these aspects related to their subjective wellbeing.

## 2. Materials and Methods 

This study consisted of two between-subject design parts. The first part was a self-administered web-based survey conducted with 385 consumers, which is the optimal sample size needed for large populations with margin of error of 5% and confidence level of 95% [45]. The second part was a food test that consisted of three groups of food-pairs conducted with 63 German consumers. Table 1 demonstrates the number, age and gender of the participants in each part of the study.

### 2.1. Self-Administered Survey

Consumers’ perception of their subjective wellbeing associated with organic food consumption and OIBG was evaluated using a self-administered survey. The survey was distributed on a European level in English, German, French, Italian and Spanish language translated by academic native speakers. The survey was shared through social media, emails and personal invitations. It considered organic food consumption in general without focusing on a specific product as consumers of organic food may vary with their preferences.

The survey consisted of multiple items derived from previous studies [7,8,46,47,48,49,50]. Some minor adjustments had been made to fit the present study. To avoid the participant’s boredom, some of the items were statements, others were questions. Table 2 shows the used items in the survey that was distributed throughout the universities’ channel, social media, emails and personal connection.

### 2.2. Food Test

According to Geier et al. [43], untrained consumers can detect changes in their mental and physical conditions consuming different types of food. The present study investigated the differences in the impact of organic and conventional food samples on consumers’ wellbeing state during a food test.

This experiment took place in the sensory laboratory of Kassel University and Fulda University of Applied Science, Germany. The test was conducted with a panel of 63 untrained German consumers, which is compatible with the minimum required number of untrained panellists to obtain statistically reliable results according to ISO 11136 [51]. The experiment was carried out under the condition of German standard DIN 10974 [52].

Consumers volunteered to take part in the experiment. They were invited to participate and received information about the study’s structure and general goals via emails, social media and personal invitations. Each participant was welcomed and had a quick chat with the experimenter to release any previous pressure or tension and to clarify the test design. During the test, participants used pre-prepared tablets to answer the questionnaire using RedJade software. The questionnaire was translated by an academic native speaker into the German language. After the test, all consumers received a small token of thanks (soft drink and flavoured yoghurt).

The experiment consists of six food tests with three groups of food stimuli. Each group consisted of a pair of samples, one organic and one conventional, Table 3. The food stimuli were chosen to be from different food categories (beverages, fruits of the same variety and bakery products), common among consumers, available in local supermarkets and with different sensory attributes. Samples that are known to have emotional effects such as coffee and chocolate were avoided.

The serving was carried out under-informed condition, a label of the organic product was displayed with the sample, in a monadic sequential design and identical conditions such as temperature (room temperature) and portion size. Stimuli groups were served in full randomization and the samples within each group were also served randomly.

As shown in Table 4, six wellbeing parameters were used as bipolar items to measure the food-related well-being status after testing each stimulus. Low values (close to one) represented a stronger connotation with the left term (e.g., light), and high values (close to five) represented a stronger connotation with the right term (e.g., heavy). These psychological parameters were adopted from a previous psychological test [43].

### 2.3. Data Analysis

RedJade sensory software was used to design the food test, randomize the serving and collect the data. Additionally, Statistical Package for Social Sciences (IBM SPSS Statistics version 24, Armonk, NY, USA) was used to conduct descriptive analysis, a Wilcoxon signed-rank test to compare the effect of organic with the conventional samples on each parameter, in addition to a Kruskal-Wallis test to investigate differences in satisfaction of life wellbeing between the categories of organic food buyers, and a Mann-Whitney U test was conducted as a post hoc test.

For the open questions, terms were searched within each question, and words with similar meanings were grouped into categories and dimensions. The number of mentions for each word and category was counted regardless of whether the same respondent mentioned the same term more than once.

Median and Inter-Quartile Range (IQR) were calculated for satisfaction and agreement scales. 

The responses scores of the satisfaction with food-related life scale (SWFL) were calculated. The score ranged from 3 (represents extreme dissatisfaction with food lifestyle) to 21 (extreme satisfaction with food lifestyle).

By measuring the purchase frequency, participants were categorized into three groups; regular buyer (consumers buy organic food more), irregular buyers (consumers buy organic and non-organic food equally) and casual buyers (consumers buy non-organic food more) based of the categorization used by Rana and Paul [5].

## 3. Results

### 3.1. Self-Administered Survey 

#### 3.1.1. Satisfaction with Life Wellbeing and Food-Related Behavior

Wellbeing was introduced to participants as the concept of overall satisfaction with life, positive emotions and mood, good physical health and social state. When participants were asked about their general satisfaction with life wellbeing, they showed a good level of (Mdn = 5, IQR = 2) satisfaction with their life wellbeing (Table 5). 

As Table 5 displays, consumers’ satisfaction on their food lifestyle was also investigated using satisfaction with food-related life scale (SWFL). The score of food-related overall satisfaction ranged from 3 (totally not satisfied with their food lifestyle) to 21 (perfectly satisfied with their food lifestyle). The average general score of consumers’ SWFL was 15. This value indicates that consumers have good satisfaction with their food behavior.

The analysis showed a statistically significant relationship between life wellbeing satisfaction and the items of SWFL (*p*-value < 0.001).

Organic food purchase frequency was measured by asking consumers about the percentage of organic food purchases from the whole food purchases. By measuring organic purchase frequency, consumers were categorized into regular buyer (consumers buy more than 50% organic food), irregular buyers (consumers that buy organic and non-organic food equally) and casual buyers (consumers that seldom buy organic food) based on the categorization used by Rana and Paul [5].

As shown in Table 6, the group of casual buyers was the biggest representative group (65.71%) among other groups of consumers. Regular buyers represented approximately one-third of the sample size (29.32%) and the smallest group size in this study was irregular buyers.

A Kruskal-Wallis test was conducted to examine the differences in satisfaction with life wellbeing according to the types of organic food buyers. Significant differences (Chi-square = 8.34, *p* = 0.016, df = 2) were found among the three categories of organic buyers (regular, casual and irregular). As presented in Table 7, Mann-Whitney showed that regular buyers differed significantly from casual buyers with their overall satisfaction with life wellbeing. However, irregular buyers had no significant differences with regular nor with casual buyers.

#### 3.1.2. How Consumers Associated Organic Food with Wellbeing Concept

When consumers were asked in the study to write the first three words that associate organic food with wellbeing concept from their perception, a total of 615 different individual words was reported. As individual words, “health” (163), “price” (45) and “sustainability” (39) were the most frequently mentioned words by consumers. All terms were grouped down into 36 categories (Appendix A), which finally were combined within five dimensions. As shown in Table 8, the food characteristics dimension was the most mentioned by consumers. The most salient individual words in food characteristics were “expensive,” “sustainable” and “organic.” Moreover, some foods, such as milk and eggs, represented the concept of wellbeing for some consumers. 

The psychological dimension was also salient (22). Organic food and wellbeing were related together with positive emotions such as happiness, satisfaction and the feeling of responsibility. 

#### 3.1.3. The Impact of Organic Food Consumption on Subjective Wellbeing

Impact of organic food behavior on consumers’ subjective wellbeing has been studied applying items adopted from previously developed scales [7,8,46,47,48,49,50]. Four dimensions (physical, emotional, social and intellectual) were adapted from previous studies [7,9,46,49] and investigate in the present work. First, consumers were asked about their perception of the impact of consuming organic food on their subjective wellbeing. Most consumers indicated agreement with the idea that organic food consumption positively affects their subjective wellbeing in general (Mdn = 5, IQR = 2). Participants also believed that organic food affects their physical health and emotions more than their social life.

Among all items within the dimensions, the “it makes me feel healthier” item received the highest value (Mdn = 6, IQR = 3). Regarding the intellectual dimension, participants had a neutral agreement on the impact of organic food consumption on the intellectual aspect of their wellbeing. Table 9 demonstrates the median and IQR values of each item of the wellbeing dimensions.

#### 3.1.4. The Intention-Behavior Gap in Organic Food Purchase (OIBG)

OIBG in consumers’ consumption was measured based on the disparity between the frequency of buying organic food in their daily life and the desired organic purchases in case of the absence of driver factors behind the gap. Eighty-six per cent of the participants stated that their organic food purchases would increase if the causes they reported (e.g., high price and lack of availability) would disappear.

Moreover, compared with the data demonstrated in Table 6, the percentage of regular buyers would increase from 29% to 76%, and the percentage of casual buyers would decrease from 66% into only 17% when the barriers behind the organic food purchase would disappear. This discrepancy between consumers daily behavior and their intentions to purchase organic food represents their OIBG.

#### 3.1.5. Consumers’ Perception of the Reasons Behind OIBG

OIBG is one of the most important issues facing the organic food market. Narrowing this gap has been receiving growing attention by researchers recently. Participants were asked to think of the times when they go to the supermarket having the intention to purchase a specific amount of organic foods but ended up purchasing less than the intended amount. Then, they were asked to list three possible reasons behind the previous scenario. A total of 712 different individual words were reported. The individual words were categorized into 11 categories. As Table 10 shows, “financial concerns” led as the most associated factor to OIBG among all other categories (268). “Availability” was the second important reason that led to OIBG (173). Consumers expressed the lack of availability using terms such as “limited choice,” “less quantity” and “seasonality.” Additionally, food characteristics (162), such as sensory attributes, quality, origin and packaging, were of the most related hinders behind OIBG. “Time pressure” during shopping was also one of the salient mentioned causes behind OIBG (31). Consumers’ negative emotions towards organic products, such as disappointment and dissatisfaction, had a role to play at the marketplace (26). “Personal characteristic” (17) and “marketing strategies” (14) were mentioned almost equally as one of the obstructers of purchasing organic food.

#### 3.1.6. Impact of Intention-Behavior Gap (OIBG) on Subjective Wellbeing

The intention-behavior gap is a state where a gap between consumers’ attitude and their behavior occurs in the marketplace. As the subjective wellbeing concept covers various aspects of life, it is important to understand how it could be affected by such a gap. 

Similar to the organic food consumption, OIBG impact on subjective wellbeing was investigated on a general level and four dimensions level (physical, emotional, social and intellectual). Consumers believed that the gap between their intentions to buy organic and their final purchase had no negative influence on their subjective wellbeing (Mdn = 4).

More than half of the participants (*n* = 385, 60%) expressed strong disagreement or disagreement on the negative impact of the OIBG on their wellbeing. As demonstrated in Table 11, the effect on the social dimension received the highest disagreement among other dimensions (Mdn = 3, IQR) = 3. The impact of OIBG on physical and emotional dimensions was perceived almost equally where participants saw no influence of the OIBG on those two dimensions. Furthermore, participants indicated disagreement on how OIBG could affect their intellectual aspect in life. 

#### 3.1.7. The Effect of OIBG on Consumers’ Emotions

The participants were asked about the effect of the gap between their intention and their behavior to buy organic food on their emotions. A total of 616 words were mentioned, of which 44 were irrelevant answers such as “price and animal welfare,” Table 12. Out of the 616 words, 455 were negative emotion words that were grouped into 49 different terms of which 35 were mentioned more than 2 times and 14 were mentioned only one time. The most salient way in which consumers expressed the OIBG influence on their emotional wellbeing was with the terms “dissatisfied” and “disappointed.” Moreover, results showed that participants expressed positive emotions after OIBG with 81 positive words that were grouped into 14 different terms. Positive terms like “relieved, well, calm and relaxed” were grouped under the term “good.” Moreover, the terms “content, delighted and cheerful” were counted under the term “happy.” Participants declared a neutral effect of the OIBG on their emotional state using 80 words such as “normal” or statements such as “I don’t know” and “I don’t care.”

### 3.2. Food Test

This study aims to understand the different effect of organic and conventional food on German consumers’ subjective wellbeing parameters during a consumption experience.

Participants were asked to test two types of food samples; organically and conventionally produced. Then, participants were instructed to rate their body changes (response) after each type of the samples. The psychological test was previously verified as a suitable method for untrained consumers to recognize changes in their state of subjective wellbeing.

Participants used bipolar scales (warm–cold, light–heavy, bright–dark, alert–tired, energized–not energized, good mood–bad mood). As Table 13 shows organic and conventional stimuli differed neither in physical nor in mental effects in general. Results revealed no significant differences between the two types of samples in any of the six tested parameters except for the perception of brightness and mood for apple juice samples, where participants felt significantly brighter and were in a better mood (*p*-value < 0.05) after drinking the organic apple juice. Additionally, after testing the organic grapes sample, participants felt brighter (*p*-value < 0.05).

Although there were no apparent significant differences between organic and conventional stimuli, consumers tended to feel warmer, lighter, brighter, more energized, more alert and in a better mood, with small differences when testing organic apple juice and grapes samples comparing to the conventional samples.

## 4. Discussion

Our work raised the question of which factors influence organic food consumption and how the intention-behavior gap may influence consumers’ subjective wellbeing. Furthermore, the work investigated the different effects of organic and conventional food stimuli on consumers’ mental and physical parameters during a food test. Plenty of prior research studied health and wellbeing as motivational factors for consumers to purchase organic food. However, few works addressed the influence of organic food consumption on subjective wellbeing [30,31]. More importantly, the potential influence of the intention-behavior gap in organic food consumption on wellbeing has not been explicitly proposed or previously studied. 

### 4.1. Self-Administered Survey

After participants were introduced to the wellbeing concept, the majority were somehow satisfied with their overall life wellbeing. Much like their satisfaction with their life wellbeing, consumers showed similar satisfaction with their food lifestyle using satisfaction with food-related life scale (SWFL). Consumers were by some means satisfied with their food behavior, which was consistent with results obtained by Grunert et al. [46]. This similarity between overall satisfaction with life wellbeing and SWFL could be linked to the significant relationship that was found between the SWFL items and the overall assessment of life wellbeing. This result highlights the importance of food as an essential part of people’s lives. A persons’ satisfaction on life wellbeing, according to the results, is related to the satisfaction with meals and food behavior. 

Moreover, the results found evidence for the relationship between satisfaction with life wellbeing and organic food behavior as regular buyers of organic food felt more satisfied with their wellbeing comparing to casual buyers. This could imply the first hint of how organic food consumption patterns have a clear relationship with the way consumers assess their life wellbeing. 

#### 4.1.1. Consumers’ Perception of the Association Between Wellbeing and Organic Food 

In previous works, when people were asked about the association of food and wellbeing, many consumers mentioned the word “organic” [7,41]. In our work, we promoted participants to think about organic food and wellbeing. “Health” was the most relevant term mentioned by consumers when thinking of the association between organic food and subjective wellbeing. In previous findings “health” was the first-mentioned aspect to link food, in general, with wellbeing [7,12,53]. In our study, we also showed that consumers’ conceptualization of wellbeing in an organic food-related context is strongly related to physical health. Thus, the present study confirms and adds to the previous findings on the association between organic food concept and “health” aspect. This result could be explained by the fact that “health,” as previously addressed, is one of the most strongly correlated aspects with the concept of organic food [12,14,39,54].

However, consumers stressed on other dimensions such as “food characteristics,” including intrinsic attributes such as “sensory attributes,” “nutritional value” and “free of pesticides,” and extrinsic attributes such as “price,” which was the second most mentioned word after “health.” This could be because consumers also link their food-related wellbeing with the economical aspect as explained by Ares et al. [7]. The fact that “food characteristic” was by far the most addressed dimension by consumers shows the importance of food attributes and organic concept to consumers concerning their wellbeing.

For some consumers, specific food (e.g., milk and eggs) also represented the relationship between organic food and subjective wellbeing which suggests the importance for those consumers to consume these specific products as organic to feel better wellbeing.

Previous works [7,55] showed that the psychological dimension is one of the most relevant dimensions in the relationship between food and wellbeing. Our results on organic food and wellbeing are consistent with the latter results. Consumers stressed positive emotions such as happiness, satisfaction and enjoyment to express their perception of organic food with regards to their subjective wellbeing. As many consumers usually show concerns about their health and feel responsible toward the environment and animal welfare, it is reasonable that consuming organic would provoke positive emotions within them feeling that they are doing good to those concerns. 

#### 4.1.2. The Impact of Organic Food Consumption on Subjective Wellbeing

We asked participants to declare their agreement on how organic food consumption affects the physical, emotional, social and intellectual aspects of their life wellbeing. Few previous works studied the general effect of organic food on wellbeing [30,31] and none of those works investigated the direct effect of organic food on different dimensions.

Consumers agreed on the general positive effect of organic food on their subjective wellbeing, which is consistent with what has been found in previous works [30,31]. Three dimensions of subjective wellbeing, emotional, physical and social dimensions were positively affected by organic food consumption. However, the intellectual dimension does not seem to be influenced by organic food consumption in the same manner as the other dimensions. Together, consumers believed that the consumption behavior of organic food positively affects their subjective wellbeing and leads to better physical state, more positive emotions and better social life. These results could be applied to improve consumers’ organic behavior. Highlighting the fact that organic behavior contributes to better personal wellbeing including not only physical but also emotional and social aspects of life would encourage consumers to consider adopting more organic food in their food lifestyle. More studies should emphasize the reward in subjective wellbeing that is related to organic food consumption to constitute a strong motive for consumers.

By showing that organic food consumption could positively impact subjective wellbeing, our work contributes to the growing global stream of studies that tackle the relationship between consumers’ subjective wellbeing and organic food consumption.

#### 4.1.3. The Intention-Behavior Gap in Organic Food Purchase

Although consumers may express high positive attitudes toward organic food, the organic food market is still suffering from the so-call intention-behavior gap phenomena (OIBG) [24,38,40,56,57,58].

As shown in our results, consumers’ purchase behavior falls short to their purchase intentions to buy organic food. This disparity is one of the real problems that are facing new calls to adopt a more sustainable lifestyle.

Consumers were asked to list three possible barriers behind the gap in their organic food purchase. It was not surprising that consumers considered by far the “financial concerns” as the first hindrance to purchasing more organic food. Specifically, consumers mentioned in addition to “expensive price” the terms “insufficient budget” and “intention to save money” preventing them from buying more expensive food. This could be strongly related to the financial status of the consumers [33,59], or it could be explained by the perception of consumers about the organic product itself. Despite its important credence values, consumers’ may perceive the organic product, as mentioned by part of the study sample, as an “unhealthy” “untrustworthy” product that does not deserve such a premium price.

Lack of availability was the second barrier in the list, as organic food has little variety or limited choices or closer expiration date comparing to other types of products. Those results are in line with the results obtained in previous studies [22,34,37,40]. Lack of availability reflects the market failure to provide organic consumers with their needs of products, which could be because this group of consumers is the least represented group in a specific market. Additionally, the lack of availability could be related somehow to high prices. If the group size of organic consumers is small in a specific market and the prices of organic products are high, which will discourage new consumers to join this group, producers and retailers would not be encouraged to supply the market with less-demanded food. Thus, the price is considered as the major reason behind OIBG.

Food characteristics also affected the final behavior of consumers. In particular, participants stressed the aspect of product quality and sensory attributes. Consumers declared that they perceive organic products as less attractive and with less quality comparing to the same products from other types.

Some participants perceived organic as unhealthy as other products, which highlights the lack of trust in the “Bio” (Organic) label and eliminates the most driven factor behind organic behavior, the “health” factor. Undeniably, the scientific significant health differences between organic diet and diets of other food types are still scarce. There has not been yet rigorous evidence that addresses the effects of organic versus conventional food on human health [60]. More studies are required to confirm or deny the validity of this belief. However, even if the motivation to buy organic came only from a psychological effect of the label, the adoption of a healthier and more sustainable lifestyle due to this effect should be still welcomed.

One notable reason of which consumers addressed was the “packaging” of the organic product. Consumers objected on the fact that organic product package may be in most cases not appropriate whether due to the package type (such as using plastic) or quantity and size (too much packaging). These results, in addition to other mentioned factors, are highly important to be taken into consideration by the organic marketers, as it tackles the search attributes that constitute the first line of the consumers’ final decision alongside the experience attributes and credence attributes of organic products. Moreover, this result could be useful for organic food marketers who mostly focus on the factors that strengthen the OIBG to avoid them for the sake of narrowing this gap.

Another noteworthy barrier, of which consumers believed that it affects their purchase behavior, is the “time” factor. Many consumers reported that they “don’t have time” during shopping whether due to the “unplanned purchase” or “lack of patience” to search and compare. Organic products are usually not visible on the shelves, and a large number of consumers see themselves forced to pay extra attention and make more efforts to find their needs [38,61]. Thus, consumers “in a hurry” try to avoid such an extra effort. Most of the research on OIBG attribute the gap to different factors such as price, availability, lack of trust, lack of knowledge, habits and overall liking [33,37,40,62,63]. However, time pressure as an influent factor in OIBG is less infrequently discussed. Time pressure is widely investigated by researchers as an important factor that influences consumers’ decision within the store [64,65,66]. It was concluded that time availability affects purchase behavior at the market place [55]. It seems that consumers try to reduce the time spent on grocery shopping, which takes less time today available for them at the supermarket. Moreover, it was proven in previous works that under time pressure, people’s behavior becomes intuitive [19,67,68,69] and they rely on their implicit attitudes [23]. This could explain why consumers, under time pressure, may tend to choose the product they are familiar with, which in most cases is the most visible product with better price, availability, quality and sensory attributes. Furthermore, consumers referred to the marketing strategies, which also could be related to the “time” barrier. Marketing strategies of organic products should be larger and stronger, the public should be informed more about the selling locations of organic food and organic products must be more visible and displayed in better locations inside the supermarkets (e.g., setting up bigger signs throughout and providing better places on the shelves). These steps could make the organic product more visible and more accessible to consumers even under time pressure.

Another barrier that was also salient, although it was mentioned in a lower frequency, is consumers’ emotions towards the product in the marketplace. Emotional aspect played a major rule in consumers’ final decision, which is consistent with previous results [7,70]. Participants declared that when they feel dissatisfied or disappointed towards the organic product, they tend not to buy it as planned. Those emotions could be driven by the search attributes of the product such as sensory attributes, the experience attributes such as a previous unpleasant experience that resulted from a bad taste or lack of trust with the credence attribute, e.g., when participants do not trust health advantages or other promoted benefits of the organic product.

#### 4.1.4. Impact of Intention-Behavior Gap (OIBG) on Subjective Wellbeing

Our work, to the best of our knowledge, is the first to cast a new light on the influence of OIBG on consumers’ subjective wellbeing considering the physical, emotional, social and intellectual aspects of their lives.

Results showed that consumers did not seem to believe that OIBG negatively impacts their wellbeing. A previous study has addressed one statement on OIBG and wellbeing. Our result concerning this statement is in line with the result obtained by this work [23]. What distinguishes the present study is that it investigated the impact of the OIBG on different dimensions of wellbeing so it can form broader comprehension of this impact. This work demonstrated that even on the dimension levels, consumers did not perceive any negative impact for OIBG on their wellbeing dimensions. Although consumers usually link organic food consumption with better health and emotions, when it comes to consuming less organic food or more conventional food, they did not perceive any negative effect for such behavior. Moreover, from consumers’ perception, OIBG does not seem to impact any of the social or intellectual aspects of their life. This has not been validated yet in organic food studies.

This is an important finding in the understanding of consumers’ perception of how the gap in their organic food behavior affects their life wellbeing. As consumers declared that consuming organic food enhanced their wellbeing, including physical, emotional and social aspects, we expected that they would perceive a negative impact of consuming less organic food than what was intended to for their wellbeing. If consumers did not believe that OIBG has any effect on their wellbeing, this may raise concerns about how to motivate consumers to narrow this gap. Alongside emphasizing the positive impact on subjective wellbeing, it is rather important to be aware of how consumers look at the effect of OIBG leaves in their lives.

#### 4.1.5. The Effect of OIBG on Consumers Emotions

The study explored the direct emotional impact of OIBG on consumers. When participants were asked using simple statements to declare their agreement on whether OIBG provokes negative emotions (It makes me feel negative emotions (e.g., unsatisfied, sad, confused) or It makes me feel bad), they declared a neutral agreement. However, when they were guided to imagine the scenario of the OIBG, they declared plenty of negative and positive emotion terms. Dissatisfied, disappointed and bad were the top provoked negative emotions by the OIBG scenario. These results show the importance of the type of questions that must be used to obtain a more comprehensive understanding of how OIBG affects consumers’ emotions. Moreover, some of the participants declared, with far less frequency, positive emotion such as good, well and relaxed. Having positive emotions toward OIBG could be a bad sign of the effort towards narrowing the gap. It is important to emphasize the provoked negative emotions to motivate consumers to be more aware of the negative impact of OIBG on this important aspect of their wellbeing.

### 4.2. Food Test

This study tried to investigate any differences between the effect of organic vs. conventional food samples on German consumers’ mental and physical wellbeing during a food test. Three groups of food stimuli (fruit, baked food and beverage), organic and conventionally grown, were used in this study. A pre-developed psychological questionnaire [43], which includes physical and mental parameters, was applied to detect the changes in consumers’ wellbeing state during food consuming experience.

Results showed that German consumers identified no significant difference between the impact of organic vs. conventional food stimuli on their subjective wellbeing parameters. Although in most cases consumers felt warmer, lighter, brighter, more alert, with more energy and in a better mood when eating organic samples, those differences between organic and conventional were very small and difficult to observe. This is in a line with the results obtained by Geier et al. [43] when they used organic and conventional produced samples among other varieties of samples in their approach. 

Regarding the so-called “label effect,” in previous work consumers declared the same emotional attitudes towards organic and conventional food samples in a blind test. Yet, in an informed test, significant differences were found between the two types of samples due to a “label effect” [23]. On the contrary, it is notable that consumers in this study were not influenced by the label as they stated their perceived effect of organic food on their mental and physical wellbeing in the same way as the conventional samples. The results demonstrated that being affected by the label effect of organic is not necessarily true in all comparisons between organic and conventional food tests. This could be explained in part by the experiment procedures. When conducting the experiment, the experimenter emphasized for each participant the importance of focusing only on their body changes. Thus, we speculate that according to the experimenter instructions, participants focused on their mental and physical body changes more than concentrating on the label. This may be the reason why we did not find an impact of the label effect.

From this standpoint, organic food intake can be considered to have the same effect on consumers’ mental and physical parameters as conventional food intake. Thus, at this stage of understanding, we believe that organic food marketers should focus more on the credence attributes of organic food-related to physical, emotional, social and other benefits (e.g., environment and animal welfare) obtained from organic consumption behavior more than focusing on the comparison with other types of food with regards to experience attributes.

## 5. Conclusions

We showed how the term “health” is the strongest term that associates organic food with the wellbeing concept, and the term “price” is the major barrier behind OIBG. Moreover, consumers in this study agreed that organic food consumption provides them with better wellbeing, including physical, emotional and social aspects. However, they did not believe that the gap between their intentions to buy organic food and their real behavior in the marketplace had any influence neither on their life wellbeing nor on the physical, emotional, social or intellectual aspects.

Furthermore, for German consumers, organic and conventional food samples during a food consumption experience had similar effects on their mental and physical wellbeing parameters of the body.

In brief, as wellbeing is a broad concept involves not only personal health but rather emotions, social and intellectual aspect of a person’s life, an important implication of this study is to boost organic food behavior by emphasizing its positive impact on subjective wellbeing; additionally, to be aware of how consumers perceive the effect of OIBG on their wellbeing. The results would impact positively organic food marketing and enhance the promotion of organic food behavior.

## 6. Limitations

The intention–behavior gap was reported in almost all European markets. The cultural effect is reported to have an impact on consumers’ perception, behavior and final choice. As the number of the sample size of different EU nationalities (and their food cultures) in this study is small and not statistically representative of the population, results must be considered as preliminary ones. Thus, a study that investigates the cultural impact on the consumers’ perception of the effect of OIBG on their wellbeing with a higher statistically representative sample is highly recommended.

Moreover, the demographic characteristics of consumers including occupation, education and income were not collected. Investigating the impact of the income, education and occupation on consumers’ perception is considered important. Moreover, studying differences between consumers’ groups based on these characteristics is also considered important. Further studies that focus on the cultural effect and involve demographic characteristics are required in the future.

## Figures and Tables

**Table 1:** Number, average age and gender percentage of the study sample.

Experiment	Number of Participants	Gender (%)		Average Age	Nationalities
Self-administered survey	385	Male	38%	48	200 German 53 French 56 Italian 23 Spanish 53 other nationalities
		Female	62%		
Food test	63	Male	40%	26	German
		Female	60%		

**Table 2:** The items used in the self-administered survey to evaluate the effect of organic food and the intention-behavior gap in organic food consumption (OIBG) on subjective wellbeing. Seven-point satisfaction scale (1: extremely dissatisfied, 4: neutral, 7: extremely satisfied) and seven-point agreement scale (1: strongly disagree, 4: neither agree nor disagree, 7: strongly agree) were applied.

Concept	Statement	Type of The Question
General introduction	Introduction statement to explain the general concept of wellbeing to make the participants familiar with the concept.	Statement
General satisfaction with life wellbeing	How much are you satisfied with your overall life wellbeing?	Seven-point satisfaction scale
Satisfaction with food-related life scale (SWFL) ^1^	-I am generally pleased (satisfied) with my food-behavior -My life is close to ideal regarding food and meals -Food and meals give me satisfaction in my daily life.	Seven-point agreement scale
Associations between organic food and subjective wellbeing	Write down the first three words that come to your mind when thinking about organic food and wellbeing.	Open-ended question
Organic food purchase frequency	How much organic food do you buy monthly? (please indicate the approximate percentage of organic purchases from your whole food purchases).	Open-ended question
The general effect of organic food on subjective wellbeing	Organic food has a good impact on my wellbeing	Seven-point agreement scale
Subjective wellbeing dimensions ^2^		
Physical dimension	-Consuming organic food has a good impact on my health -It helps me feel healthier	Seven-point agreement scale
Emotional dimension	-It makes me have positive emotions (e.g., proud, satisfied, happy) -It makes me feel good	Seven-point agreement scale
Social dimension	-It makes me feel more connected to surrounding people -It improves my self-image in front of others	Seven-point agreement scale
Intellectual dimension	-Consuming organic food helps me have a purposeful and meaningful life -It reflects more my self-knowledge and beliefs	Seven-point agreement scale
Drivers behind OIBG	What are the first three reasons that come to your mind when thinking about the gap between what you planned and what you purchased?	Open-ended question
The effect of OIBG on consumers’ feelings and emotions	How does this gap make you feel?	Open-ended question
The effect of OIBG on consumers’ subjective wellbeing	This gap has a negative impact on my well-being	Seven-point agreement scale
Physical dimension	-It makes me feel less healthy -It negatively influences my health	Seven-point agreement scale
Emotional dimension	-It makes me feel negative emotions (e.g., unsatisfied, sad, confused) -It makes me feel bad	Seven-point agreement scale
Social dimension	-It affects negatively my image in front of surrounding people -It makes me feel disconnected to the surrounding society	Seven-point agreement scale
Intellectual dimension	-It affects negatively my values in life -It influences badly my improvement in life	Seven-point agreement scale
Organic food purchase intention	If we supposed that all the obstacles you perceive to provoke the OIBG were overcome, how much organic food would you buy then? (please indicate the percentage from your whole monthly purchases)	Open-ended question

^1^ Items were adapted from [45]. ^2^ Items were adapted from [7,9,46,49].

**Table 3:** The product information on the six food stimuli.

Product Category	Product	Brand/Origin	Description
Beverage	Apple juice	Alnatura	Direct juice, red apples, cloudy juice, organically produced
		Tegut	Direct juice, red apples, cloudy juice, conventionally produced
Fruits	Fresh grapes	Origin: Italy	Organic seedless grapes
		Origin: Italy	Conventional seedless grapes
Bakery product	Spelt toast bread	Herzberger Bio-Bäckerei	Organic Dinkel wheat toast bread
		Golden toast	Conventional Dinkel wheat toast bread

**Table 4:** The six bipolar items, rated on five points, that were used as the wellbeing parameters to measure consumers’ food-related wellbeing.

When I Test This Sample	English		German	
my body feels	warm	cold	warm	kalt
	light	heavy	leicht	schwer
	bright	dark	hell	dunkel
I feel	alert	tired	erfrischt	müde
	energized	not energized	energetisiert	nicht energetisiert
my mood becomes	good	bad	Gut	schlecht

**Table 5:** The median and Inter-Quartile Range (IQR) of consumers’ overall satisfaction with life and satisfaction with food-related lifestyle (1 = strongly disagree, 7 = strongly agree) and (1 = strongly dissatisfied, 7 = strongly satisfied).

Concept	Median	IQR
Overall wellbeing satisfaction	5	2
Satisfaction with food-related life	5	2
	5	2
	6	2

**Table 6:** Percentage of consumers group based on their organic food purchase and organic food purchase intentions. The consumers were categorized into regular buyers who buy/intend to buy organic food more than other types of food, casual buyers who buy/intend to buy organic food less than other types of food and irregular buyers who buy/intend to buy organic food equally as other types of food.

The Group of Organic Food Buyers	% Out of the Study Sample (*n* = 385)	
	Purchase behavior	Purchase intention
Regular	29.0%	75.8%
Irregular	5.5%	8.0%
Casual	65.5%	16.2%

**Table 7:** Mann-Whitney analysis shows that the differences in satisfaction with life wellbeing between the different categories of organic food buyers. * Significance at *p* < 0.05.

Regular vs. Casual *		*p*	Regular vs. Irregular		*p*	Casual vs. Irregular		*p*
Mean rank (regular)	Mean rank (casual)	0.014 *	Mean rank (regular)	Mean rank (irregular)	0.374	Mean rank (casual)	Mean rank (irregular)	0.065
201.26	173.3		66.74	74.45		133.98	165.09	

**Table 8:** Frequency of mention of the five dimensions of organic food-related wellbeing and the most salient individual words when participants were asked to write down the first three words that come to their mind when thinking about organic food and subjective wellbeing.

Dimension	Individual Words	Number of Mentions
Characteristics of food	Intrinsic attributes: taste (tasty and not tasty), free of chemicals/poisons/toxins/pesticides, nutritious, GMO-free, green, fresh, delicious, natural Extrinsic: expensive, sustainable, organic, label, safe, quality, clean, ethical, ecology, genuine, normal, brand (Demeter), good, eco-friendly, good for future, variety, diversity, great, transparent, real, new or fashion, cool, controlled, certified, trustful, good for animal welfare, bulk food, fair trade, food origin: regional/European/local/rural, harmful for the environment, untrustworthy, not sustainable	375
Physical health	Health, wellness, wellbeing	163
Specific food	Milk, eggs, salad, dry fruits, cucumber, apples, protein, meat, juice, peanut butter, organic fruits, vegetables, potatoes, tomatoes, onion	25
Psychological aspects	Happiness, satisfaction, invigorating, enjoyment, responsibility, positive feelings, emotionally balanced, pleasant, security, serenity, tranquillity	22
Personal attributes	Knowledge, awareness, education	17
	Life standards, organized life, long life, better life, accomplishments, lifestyle, childhood	8
	Less meat, no meat, vegetarian, vegan	5

**Table 9:** The median and IQR values of the nine items used to study organic food impact on subjective wellbeing (1 = strongly disagree, 7 = strongly agree).

Concept	Item	Median	IQR
The general effect of organic food on subjective wellbeing	Organic food has a positive impact on my wellbeing	5	2
Physical dimension	Consuming organic food has a good impact on my health	5	2
	It helps me feel healthier	6	3
Emotional dimension	It makes me have positive emotions (e.g., proud, satisfied, happy)	5	2
	It makes me feel good	5	2
Social dimension	It makes me feel more connected to surrounding people	5	2
	It improves my self-image in front of others	5	2
Intellectual dimension	Consuming organic food helps me have a purposeful and meaningful life	4	2
	It reflects more my self-knowledge and beliefs	4	3

**Table 10:** Frequency of mention of the most individual words and categories when participants were asked to write down the first three factors behind the intention-behavior gap during their organic food purchase.

Category	Items	Number of Mention
Financial concerns	Expensive, insufficient cash, economic, financial aspects, limited budget, thrift, intention to save money	268
Availability	Little variety, lack of organic version, less quantity, little choice, seasonal products, scarcity, expiration date, lack of the wanted brand, lack of unpacked organic products, lack of presence, limited quantity	173
Food characteristics		162
	Quality	47
	Origin (European, non-European, local, regional)	27
	Sensory attributes: unappetizing appearance, taste, flavour, unattractiveness	25
	Packaging (size, type of packaging such as plastic package, too much packaging)	18
	Information	15
	Not healthy	13
	Variety	8
	Convenience	8
	Utility	1
Time	Have no time, having no patience to search, unplanned purchase, impulsiveness, spontaneous buying decision, sudden hunger (so the purchase is done too fast), being in a hurry	31
Emotions	Disappointment, dissatisfaction, frustration, distrust, discomfort, cumbersome, nervousness, confusion	26
Personal characteristics	Lack of knowledge, mismanagement, forgetfulness, haste, laziness, inattentiveness, lack of self-control, inconsistency	22
Marketing	Promotion discount, advertising, location at the store, competition with other types of products	14
I do not know		7
Social effect	Roommates, consumers within the family, family habits, culture and believes	5
Food habits	Eating unhealthy, food habits	4

**Table 11:** The median and IQR of the nine items that were used to study the impact of the intention–behavior gap in organic food consumption (OIBG) on subjective wellbeing (1 = strongly disagree, 7 = strongly agree).

Concept	Items	Median	IQR
The general effect of OIBG subjective wellbeing	This gap has a negative impact on my well-being	4	
Physical dimension	It makes me feel less healthy	4	2
	It negatively influences my health	4	2
Emotional dimension	It makes me feel negative emotions (e.g., unsatisfied, sad, confused)	4	2
	It makes me feel bad	4	2
Social dimension	It affects negatively my image in front of surrounding people	3	3
	It makes me feel disconnected to the surrounding society	3	3
Intellectual dimension	It affects negatively my values in life	4	3
	It influences badly my improvement in life	4	3

**Table 12:** Frequency of mention of the emotion terms that were reported by the participants when they were asked to state the first three emotions that come to their mind when they think of how the intention-behavior gap makes them feel.

Emotion Term	Number of Mentions	Emotion Term	Number of Mentions
Negative Emotions		Damaged	3
Dissatisfied	73	Confused	3
Disappointed	50	Unhealthy	3
Bad	45	Bored	2
Frustrated	23	Defeated	2
Guilty	20	Insecure	2
Sad	20	Neglected	2
Annoyed	14	Sorry	2
Other negative emotions ^1^	14	Pretentious	2
Unhappy	12	Defeated	2
Poor	11	Undecisive	2
Restricted	11	Positive Emotions	
Worried	12	Good	34
Ashamed	9	Satisfied	10
Helpless	8	Healthy	11
Inconsistent	8	Motivated for next time	9
Uncomfortable	8	Great	4
Demotivated	7	Clean	2
Critical	7	Cool	2
Angry	6	Happy	7
Doubtful	4	Other positive emotions ^2^	6
Nervous	4	Neutral	
Upset	4	Normal	60
Wrong	4	I don’t know	12
Stingy	4	I don’t care	8
Undisciplined	4	Irrelevant terms	44

^1^ Other negative emotion terms: careless, lazy, weak, drained, disengaged, misled, hopeless, broke, anguished, fatigue, irritated, unforgiving, stupid, indecisive. ^2^ Other positive emotions: peace, interesting, balanced, positive, useful, proud.

**Table 13:** Mean values for each criterion of the bipolar scale of 1 (warm, light, bright, alert, energized, good mood) to 5 (cold, heavy, dark, tired, not energized, bad mood) for the three groups of samples (organic and conventional). Perc. stands for perception.

		Perc. of Warmth	Perc. of Lightness	Perc. of Brightness	Perc. of Alertness	Perc. of Energy	Mood
Spelt bread	Organic	2.74	2.98	2.94	3.12	3.27	3.04
	Conventional	2.90	2.99	2.79	3.02	3.42	2.97
Apple juice	Organic	2.84	2.34	2.04	1.97	2.06	1.94
	Conventional	3.10	2.36	2.28	2.22	2.31	2.28
Grapes	Organic	2.86	1.92	2.03	2.02	2.21	1.93
	Conventional	3.11	2.08	2.23	2.08	2.34	2.22

## Appendix A

**Table A1:** Frequency of mention of the 36 categories of organic food-related wellbeing and the most salient individual words when participants were asked to write down the first three words that come to their mind when thinking about organic food and subjective wellbeing.

Category	Number of Mentions
Health	163
Price	45
Sustainability	39
Organic	38
Pesticide-free	35
Eco-friendly	31
Natural	25
Specific food	25
Positive emotions	22
Good	21
Knowledge	17
Better	14
Quality	13
Green	10
Safe	10
Others	10
Trust	9
Certificate	7
Clean	7
Fresh	7
Tasty	7
Animal welfare	7
Nutritious	6
Bad	6
Lifestyle	6
Untrustworthy	6
Food diet	5
Fair	5
Ethical	4
Region	4
Genuine	3
Normal	2
Ecology	2
Childhood	2
GMO-free	1
Brand	1
